# Tool selection and the ventral‐dorsal organization of tool‐related knowledge

**DOI:** 10.14814/phy2.13078

**Published:** 2017-02-09

**Authors:** Michael J. Tobia, Christopher R. Madan

**Affiliations:** ^1^ Department of PsychologyWake Forest UniversityWinston SalemNorth Carolina; ^2^ Center for Mind/Brain Sciences (CIMeC)University of TrentoTrentoItaly; ^3^ Department of PsychologyBoston CollegeChestnut HillMassachusetts

**Keywords:** IPL, IPS, MTG, praxic knowledge, semantic knowledge, tool use

## Abstract

Tool selection is a cognitive process necessary for tool use, and may rely on distinct knowledge under different conditions. This fMRI experiment was designed to identify neural substrates mediating tool selection under different conditions. Participants performed a picture‐matching task that presented a recipient object and an action‐goal, and required the selection of the best tool object from among four candidates. Some trials allowed selection of the prototypical tool, whereas others forced selection of either a functionally substitutable or impossible tool. Statistical contrasts revealed significantly different activation between *Proto* and *Sub* conditions in frontal, parietal, and temporal lobes. The middle temporal gyrus (MTG) bilaterally, and the right posterior cingulate were more strongly activated by prototypical tool selection, and left inferior parietal lobule (IPL), intraparietal sulcus (IPS), middle frontal gyrus, and precuneus were more strongly activated when selecting substitutable objects. These findings are concordant with previous neuroimaging studies of tool use knowledge in demonstrating that activation of the MTG represents functional knowledge for conventional tool usage, and activation of the IPL/IPS supports action (i.e., praxic) knowledge representations. These results contribute to the literature that dissociates the roles of ventral and dorsal streams in tool‐related knowledge and behavior, and emphasize the role of the left hemisphere for processing goal‐directed object interactions.

## Introduction

Tools are objects that facilitate interactions with other objects (i.e., recipients) in the service of goal‐directed behavior. Research suggests that tool‐related processing can be divided into two general domains of knowledge: action and function. Knowledge of an object's action‐related properties relate to how an object can be manually (i.e., motorically) manipulated, and is related to representations in a dorsal pathway through the parietal cortex (e.g., Boronat et al. 2005; Canessa et al. 2008; Almeida et al. 2010). Knowledge of an object's functional properties relate to conceptual knowledge such as its typical use (e.g., Canessa et al. 2008; Ishibashi et al. 2011; Madan and Singhal 2012a; Peelen and Caramazza 2012; Fairhall and Caramazza 2013; Garcea et al. 2013), rather than motoric knowledge of its manual manipulation, and is associated with a ventral pathway through the temporal cortex. Proper tool selection is necessary for successful tool use, and requires both types of knowledge in order to correctly alter the state of the recipient object, but the degree to which one domain contributes to tool selection may vary depending on the assortment of candidate objects from which to choose and use as a tool for a particular goal.

Proper tool selection involves the integration of information concerning the recipient object whose state is to be altered with the action‐goal that prescribes a successful change of state. If the action‐goal is to stir coffee and the recipient object is a cup of coffee, then selection of a prototypical tool (i.e., spoon) is a relatively easy and appropriate choice. However, when the prototypical tool is not available, an alternative object may be substituted if its structural properties sufficiently afford its use to alter the state of the recipient object in a similar manner as the prototypical tool. For instance: If a spoon is not available, but a carrot is available as a candidate from which to choose, the structural features of the carrot sufficiently afford the accomplishment of stirring coffee. As such, selecting a prototypical tool and deciding whether an alternative object is sufficiently substitutable for the prototypical tool require access to different types of tool‐related knowledge.

Action and function knowledge are two dissociable domains of tool‐related knowledge. Action knowledge is related to how an object can be motorically manipulated, such as its graspability, and is associated with the structural properties of the visual object. For instance, if an object has a long‐axis, such as a screwdriver or carrot, this structural property provides action‐related information related to how the object can be grasped (Almeida et al. 2010; Creem‐Regehr and Lee 2005; Guérard et al., 2015; Sakuraba et al. 2012). Alternatively, a scissors and pliers are motorically manipulated in a similar manner, but have different functions (i.e., cutting vs. gripping). Function knowledge of a tool is related to the purpose or goal of tool use (Kellenbach et al. 2003; Garcea and Mahon 2012; Madan and Singhal 2012a,b; Almeida et al. 2013; Madan et al. 2016), and is associated with the semantic properties of the visual object, rather than its structural properties per se; a scissors and a knife serve related functions (i.e., cutting), but are used with different actions.

Action and function tool‐related knowledge are mediated by two different pathways in the brain, analogous to the ventral/dorsal distinction for visual perceptual processing, in which semantic knowledge about visual objects (i.e., relation to other objects) preferentially activates the ventral pathway, and perceptual features of visual objects (i.e., size, shape) preferentially activates the dorsal pathway (Milner and Goodale 2008). The action domain of tool‐related knowledge, including knowledge of graspability and motoric gesturing, is associated with a dorsal pathway, including regions such as the inferior parietal lobule (IPL), intraparietal sulcus (IPS), and premotor cortex (Kellenbach et al. 2003; Boronat et al. 2005; Canessa et al. 2008; Almeida et al. 2010; Sakuraba et al. 2012; Buxbaum et al. 2014; Chen et al. 2016). The function domain of tool‐related knowledge is associated with a ventral pathway, including the middle temporal gyrus (MTG) as well as inferior and anterior temporal cortex (Canessa et al. 2008; Ishibashi et al. 2011; Peelen and Caramazza 2012; Fairhall and Caramazza 2013; Chen et al. 2016). The posterior MTG in particular is associated with category‐selective visual perceptual processing of tools and tool‐related motion (Chao et al. 1999; Chao and Martin 2000; Beauchamp et al. 2002, 2003; Noppeney et al. 2006; Perini et al. 2014), while more anterior regions of the MTG are recently associated with knowledge about a tool's use, such as tool‐recipient matches and mismatches (Mizelle and Wheaton 2010a,b), and other tool‐related semantic knowledge (Ishibashi et al. 2011).

The purpose of this experiment was to investigate whether tool selection differently activates the dorsal and ventral neural pathways in association with differential demands for action and function knowledge when choosing a tool from a constrained assortment of objects as candidates. Participants were scanned with fMRI while they performed a picture matching task during which they were presented with a recipient and action goal, along with four objects from which to select the one that would best accomplish a specified goal. Some trials allowed selection of the prototypical tool for the specified recipient/action‐goal pair, whereas other trials forced selection of either a functionally substitutable or impossible tool. This design dissociates action and function tool‐related knowledge during the tool selection process because only the availability of objects from which to choose differs between conditions, whereas the action‐goal and target recipient are fixed.

## Methods

### Participants

A total of 17 healthy right‐handed volunteers (age: *M *=* *28.5 years old, range = 22–52; 7 female) provided written informed consent and were paid 15€ for participation. A routine medical examination prior to participation screened for a history of neurological or psychiatric disorders, and contraindications to MRI scanning. The experimental procedure was approved by the local research ethics board and all protocols were carried out in accordance with the Declaration of Helsinki. Data from one participant were excluded because they reported falling asleep during the experiment.

### Tool selection task

**Figure 1:**
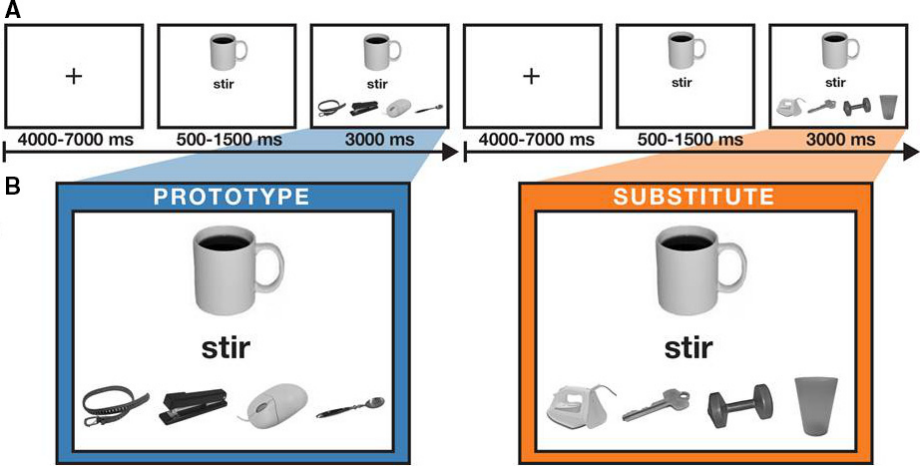
Tool selection task screenshots.Panel A illustrates a sequence of screen shots from the actual task used in the experiment, and indicates the timing and duration of each stimulus event. Panel B shows examples of stimuli that were used as the decision context for *Proto* and *Sub* trials (*Imp* not shown). The left image shows an example of the prototypical tool for stirring coffee (i.e., a spoon), and an example of a substitutable object such as a key (for stirring coffee). Additional stimuli that cannot be used to accomplish the action goal are also present on each trial as distractors. Example stimuli are from Brodeur et al. (2014).

The tool selection task presented participants with an image of a recipient object, action instruction, and an array of four available objects from which to select which would best allow accomplishment of the designated action. The recipient object appeared at the top of the display and the action‐goal appeared below it. Participants held a one‐two‐button response box in each hand and pressed buttons with their index and middle fingers to indicate which object (i.e., tool) would allow them to complete the specified action on the recipient object. Figure 1A illustrates a sequence of screenshots from the task. The cue (recipient/action‐goal pair) appeared in the top half of the display and remained visible for the duration of the trial. The assortment of the four potential tool/objects appeared after a 500–1500 msec delay, for a 3000 msec duration, during which time participants responded via button press. The display was then replaced by a fixation cross at the center of the screen until onset of the next trial (ITI = 4000–7000 msec).

The task consisted of 108 total trials, with three different trial types, based on the available object that best accomplished the designated action: prototype (*Proto*), substitute (*Sub*), impossible (*Imp*). The three recipients were a coffee cup, a screw that was driven halfway into a plank of wood, and a small pile of dirt (see Fig. 1B). Action instructions requiring tool‐mediated manipulation were ‘*stir’*, ‘*turn’*, and ‘*sweep’* for the coffee cup, woodscrew, and dirt pile recipients, respectively. Six runs of 24 randomly intermixed trials were created in which the recipient was constant within a run but the assortment of object choices changed (i.e., included a prototypical tool, substitutable object, or no objects that could accomplish the goal). There were 2 runs for each recipient object, and the order of the six runs was randomized across participants. Each *Proto* trial included a unique image of the prototypical tool; each *Sub* trial included a different sufficiently substitutable object with no repetitions. Distracter tools/objects (36 *Proto*; 44 *Sub*) appeared with random repetition on *Proto* and *Sub* trials throughout the task, and also appeared on *Imp* trials, although no configuration of choice stimuli was repeated. For some trials, objects which were correct *Sub* responses in one condition appeared as distracters in another condition, and several objects were designated as correct *Sub* responses for two conditions (e.g., a butter knife can be used to stir coffee or turn a flat‐head screw, but in neither case is it the prototypical tool).

Prior to the experiment, participants were shown a series of example slides for each trial type and demonstrated their comprehension of the task instructions to the experimenter by pointing to the correct response and pantomiming the appropriate action. Participants completed 20 practice trials of the computerized task to become familiar with the response buttons held in each hand, and asked questions about the task to remedy any remaining confusion. The experimenter acknowledged that some trials would be more difficult than others and instructed participants to select a response on every trial.

### MRI data acquisition

Magnetic resonance (MR) data were acquired with a 4 T Bruker MedSpec whole‐body MRI (Bruker GmbH; Ettlingen, Germany) with an 8‐channel head coil. High‐resolution anatomical images were acquired with a 3D T1‐weighted MPRAGE (1 mm^3^ isotropic voxels; 256 × 224; 176 slices; TR/TE = 2700/4 msec; 7^o^flip angle). Functional BOLD images covered the whole‐brain volume and were acquired with an ascending‐interleaved single‐shot GE‐EPI sequence (TR/TE = 2000/30 msec; flip angle = 12^o^; anterior‐posterior phase‐encode direction; distance factor = .15; 64 × 64 matrix; 36 slices; 3 mm^3^ isotropic voxels). In addition, a point‐spread function was collected to correct for image distortion of geometry and intensity caused by the high strength magnetic field (Zaitsev et al. 2004).

### Data analysis

Mean response time (RT) and accuracy were calculated within participants for the *Proto* and *Sub* conditions. Data from the *Imp* condition were not analyzed for accuracy as there was no correct response, by definition. A *χ* ^*2*^‐frequency analysis was used to determine the lower‐bound for accuracy that would be significantly above chance (25%) performance, which was found to be 34%. Participants whose overall task performance was below this level were excluded from further analyses (*N *=* *0).

Preprocessing and analyses of MRI data were completed, using AFNI and SUMA (Cox 1996; Saad et al. 2004). Functional image preprocessing included slice timing correction, volume registration, detrending, and removal of variance related to head movements performed separately for each scanning run with a linear regression in the volume, followed by projection to reconstructed cortical surfaces (Argall et al. 2006), and spatial smoothing on cortical surfaces to a target 6‐mm FWHM with heat‐kernel smoothing (Chung et al. 2005). Cortical surface reconstruction was performed using FreeSurfer 5.1 (https://surfer.nmr.mgh.harvard.edu/).

A predictor time‐course was created for each of the three trial types (*Proto*,* Sub*,* Imp*) using the onset of the choice assortment for each trial and convolving it with a canonical hemodynamic response function. Separate regressors for left‐ and right‐handed button presses were also included, as well as a regressor for error trials. Error trials were modeled separately to reduce both false‐positive and false‐negative activation clusters (Murphy and Garavan 2004). In addition, there were two (left/right hand) RT amplitude modulated regressors to account for trial‐by‐trial differences in RT for all trials (Grinband et al. 2008). Time points with >0.5‐mm head movement were censored from analyses (<0.1% of volumes). Second‐level group analyses were conducted on the cortical surfaces with a series of *t*‐tests to contrast activation between conditions. Second‐level analyses were cluster size thresholded on the cortical surface determined by Monte Carlo simulations to a whole‐brain corrected *P *<* *0.05 (*t *>* *3.73, *P *<* *0.001, surface radius of 4‐mm covering an area of 252 mm^2^). A minimum statistic conjunction null (MSCN) analysis (Nichols et al. 2005) to identify activation that was common to both the *Proto* and *Sub* conditions was computed as the intersection of thresholded group maps for each condition (single voxel *P* < 0.005) that survived a minimum of 100 mm^2^ cluster area.

## Results

### Tool selection task performance

**Figure 2:**
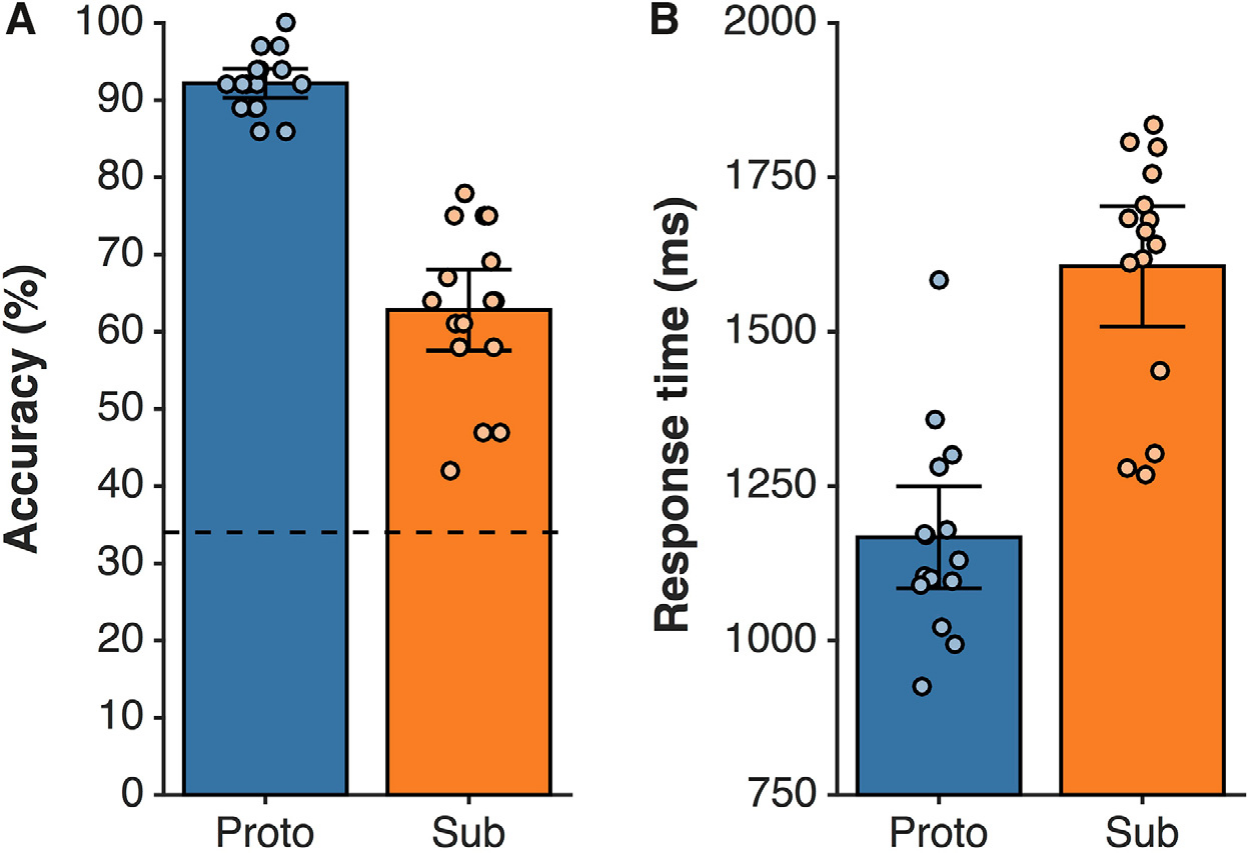
Behavioral task performance characteristics for each participant. Panel A shows group‐level task average accuracy for *Proto* and *Sub* trials (blue and orange bars, respectively), along with average accuracy for each participant (circles). The dashed line indicates the level of chance performance over the entire task. All participants overall task performance exceeded this threshold. Panel B shows group‐level average response times for *Proto* and *Sub* trials (blue and orange bars, respectively), along with each participant's RT (circles). All participants’ individual data showed a consistent relationship of greater response time for *Sub* trials as illustrated in the group level averages.

Figure 2A shows that task accuracy for all participants surpassed the threshold for greater than chance performance. Most participants achieved greater than chance performance for each condition (see Table 1). Performance for the *Sub* trials was most accurate for the Stir:Coffee condition (100% at the threshold or greater than chance), and was least accurate for the Sweep:Pile condition (85% at the threshold or greater than chance). Over the entire task, mean accuracy for *Proto* was significantly greater than *Sub* [*M*_*proto* _= 92.2%, *M*_*sub*_^* *^= 62.8%; *t*(15) = 11.75, *P *<* *0.001, *d *=* *1.74]. Participants were able to correctly identify the prototypical tool for the specified action‐goal and recipient for more than 90% of trials, and they were able to choose the correct experimenter defined substitutable object for greater than 63% of trials.

**Table 1:** Tool selection task accuracy for each condition

Subject ID	Stir:coffee		Turn:screw		Sweep:pile	
	*Proto*	*Sub*	*Proto*	*Sub*	*Proto*	*Sub*
1	.83	.92	1.00	0.67	0.92	0.33
2	1.00	1.00	1.00	0.75	1.00	0.25
3	1.00	1.00	1.00	0.75	0.92	0.50
4	0.92	0.92	0.83	0.92	0.92	0.42
5	0.92	0.75	1.00	0.33	0.83	0.33
6	1.00	0.83	0.92	0.75	0.92	0.75
7	1.00	0.83	1.00	0.58	0.75	0.42
8	0.83	0.33	0.92	0.58	0.83	0.33
9	1.00	0.75	0.92	0.75	0.75	0.42
10	1.00	0.50	0.92	0.67	0.92	0.25
11	1.00	0.83	1.00	0.67	0.83	0.58
12	1.00	0.92	0.92	0.50	0.75	0.33
13	1.00	0.58	0.92	0.67	0.67	0.50
14	1.00	0.83	0.92	0.67	0.83	0.42
15	1.00	0.92	1.00	0.92	0.75	0.42
16	1.00	0.83	1.00	0.67	0.92	0.33
*M*	0.97	0.80	0.95	0.68	0.84	0.41

Figure 2B displays each participant's RTs for the *Proto* and *Sub* conditions. *Proto* trials were consistently the most rapid responses, with *Imp* trials consistently the slowest responses. Over the entire task, mean RT for *Proto* was faster than *Sub* [*RT*_*proto*_^* *^= 1167 msec (±132.45 msec); *RT*_*sub*_ = 1606 msec (±311.6 msec); *t*(15) = 13.07, *P *<* *0.001, *d *=* *2.54], indicating that participants were able to identify the correct prototypical tool more rapidly than they were able to choose a substitutable object, which required on average 500 msec more to make a correct selection. The group mean and standard deviation for RT for the *Imp* condition was 1922.59 msec (±299.59 msec), which is significantly longer than group means for both *Proto* and *Sub* trials. This suggests that participants were indeed engaged with the problem presented on each *Sub*/*Imp* trial, and recognized after some deliberation on *Imp* trials that there was no correct response, and emitted a guess or random error response. In combination, these results show that participants were indeed processing the demands of the tool selection task, the *Sub* condition was more challenging than the *Proto* condition, and that participants were able to solve the task on a significant portion of the *Sub* trials.

### Neuroimaging results

**Figure 3:**
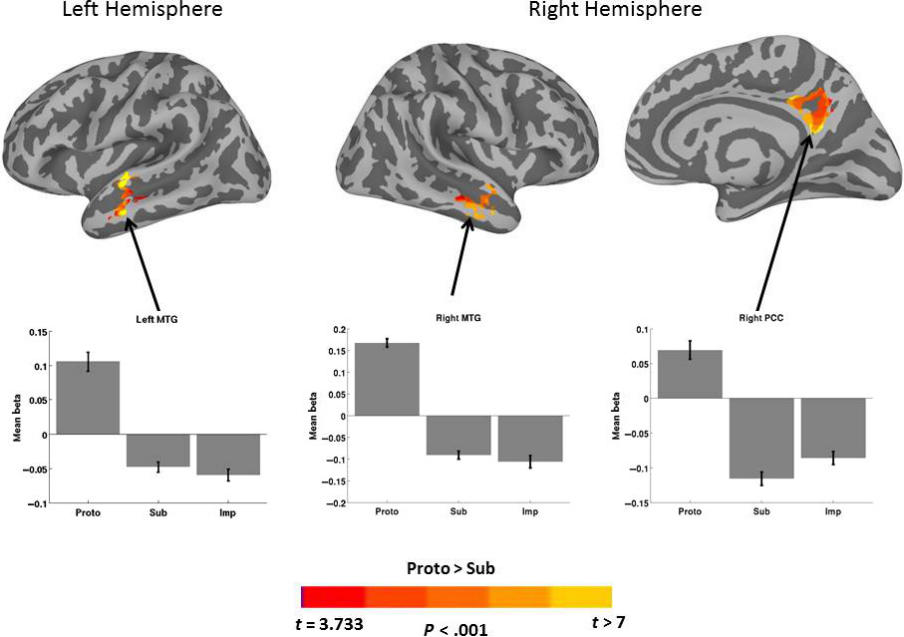
Significant activation differences for the contrast *Proto* greater than *Sub*.Cortical surface renderings are overlaid with the results of paired *t*‐tests contrasting *Proto* and *Sub* conditions. The MTG is more strongly activated for the *Proto* condition versus the *sub* condition, bilaterally, and also included stronger activation of the right PCC. The bar graphs show the average beta from each significant cluster for the *Proto*,* Sub* and *Imp* conditions from left to right. The clusters shown in this figure all indicate positive beta values for the *Proto* condition, and negative beta values for the *Sub* and *Imp* conditions. Arrows point to the approximate locations of the peak coordinates listed in Table 2. Error bars indicate standard error of the mean. MTG, middle temporal gyrus; PCC, posterior cingulate cortex.

**Figure 4:**
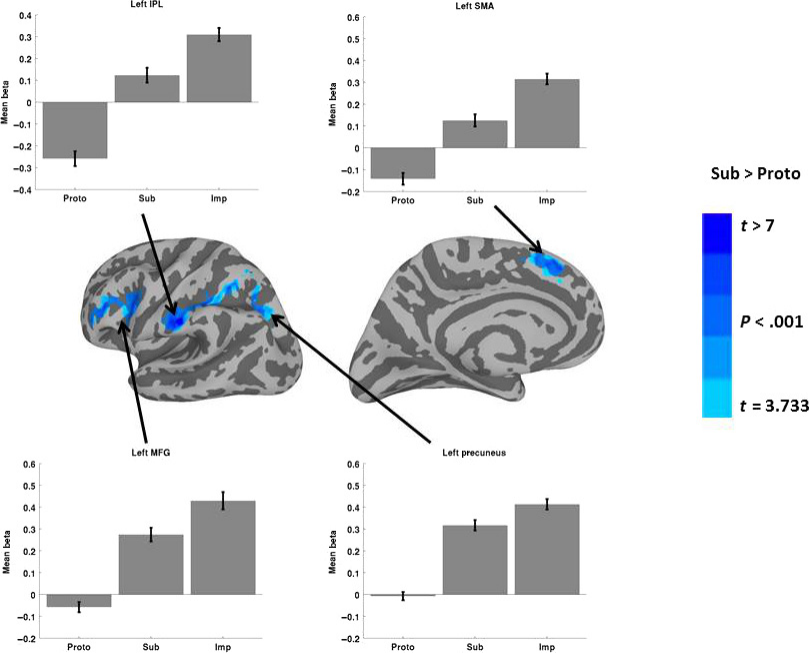
Significant activation differences for the contrast *Sub* >*Proto*. Cortical surface renderings are overlaid with the results of paired *t*‐tests contrasting *Sub* and *Proto* conditions. A large cluster of activation encompassing the IPL and IPS in the left hemisphere is shown that was significantly more strongly activated for the *Sub* condition versus the *Proto* condition. The peak voxel for the contrast was located at the IPL, although the activation clearly extends into the IPS with a peak at the precuneus, and also involved the middle frontal gyrus and supplementary motor area (medial surface). The bar graphs show the average beta from each significant cluster for the *Proto*,* Sub* and *Imp* conditions from left to right. The clusters shown in this figure all indicate positive beta values for the *Sub* and *Imp* conditions, and negative beta values for the *Proto* condition. Arrows point to the approximate locations of the peak coordinates listed in Table 2. Error bars indicate standard error of the mean. MFG, middle frontal gyrus; IPL, inferior parietal lobule; SMA, supplementary motor area.

Table 2 presents MNI coordinates for significant clusters resulting from each contrast or conjunction analysis reported below. Figure 3 shows results for the *Proto*>*Sub* contrasts. Only one cluster in the left hemisphere with a peak single voxel *t*‐value at the MTG, and two clusters in the right hemisphere with peaks at the MTG and the posterior cingulate cortex (PCC), survived the threshold for whole brain correction. The clusters whose peaks were located at the MTG also extended into the superior temporal sulcus (STS) and the superior temporal gyrus (STG), showing that the anterior temporal cortex was more involved for prototypical tool selection than choosing a substitutable object. The graphs illustrate the sign of the relationship between activity in a particular cluster and the hemodynamic predictor time course. *Proto* trials showed a positive relationship of the predictor time course for each of the three clusters, whereas *Sub* and *Imp* trials showed a negative relationship. Figure 4 shows results for the *Sub*>*Proto* contrast. Four clusters in the left hemisphere demonstrated greater activation at the IPL, precuneus in posterior IPS, middle frontal gyrus (MFG), and supplementary motor area (SMA). No clusters survived the cluster threshold in the right hemisphere for the *Sub*>*Proto* contrast. In addition, the graphs indicate that these clusters show the opposite signed relationship to the predictor time courses for *Proto*,* Sub* and *Imp* trials as shown in Figure 3. *Sub* and *Imp* trials showed a positive relationship, and *Proto* trials showed a negative relationship. This shows that *Sub* and *Imp* trials are engaging in similar neural resources, and that *Proto* trials recruit a different set of neural resources. Together, these results demonstrate that tool selection differently activates portions of the ventral and dorsal pathways dependent on whether the prototypical tool is available or not.

**Table 2:** Activation clusters

	Peak MNI: x y z	Peak *t*‐val	Area(mm^2^)
Proto > Sub			
Left MTG	−59 −16 −16	8.40	256.58
Right MTG	57 −7 −9	9.35	463.75
Right PCC	8 −50 28	7.20	280.98
Sub > Proto			
Left IPL	−47 −35 38	10.04	808.34
Left Precuneus	−25 −62 35	6.90	563.16
Left MFG	−42 12 28	7.19	527.69
Left SMA	−9 24 48	6.78	268.17
Proto:Sub Conjunction			
Left Insula	−34 1 17	6.57	396.65
Left Inferior OccG	−41 −82 −17	5.56	210.61
Left Cuneus	−5 −92 22	5.80	189.30
Left Lingual Gyrus	−15 −51 −1	5.71	128.30
Left Lingual Gyrus	−2 −73 0	6.91	117.70
Right MidOccG	24 −98 −2	5.87	187.01
Right Fusiform Gyrus	44 −67 −17	7.50	177.20
Right Insula	37 −7 1	5.03	138.69
Right IPL	62 −25 31	5.51	112.51
Imp > Proto			
Left MFG	−39 31 20	8.74	2710.24
Left Precuneus	−23 −61 41	10.95	2552.54
Left medialFG	−8 16 51	10.13	1457.41
Left Cuneus	−20 −73 3	7.06	691.44
Left MOccG	−45 −62 −12	7.83	475.55
Right Lingual Gyrus	23 −63 2	9.73	962.56
Right Insula	29 24 5	9.76	794.60
Right Cingulate Gyrus	8 23 43	9.40	599.70
Right Precuneus	27 −62 33	6.39	303.81
Proto > Imp			
Left MTG	−64 −21 −16	7.85	560.49
Right PCC	9 −41 32	9.19	550.46
Right MTG	62 −14 −17	11.54	273.62

IPL, inferior parietal lobule; SMA, supplementary motor area

**Figure 5:**
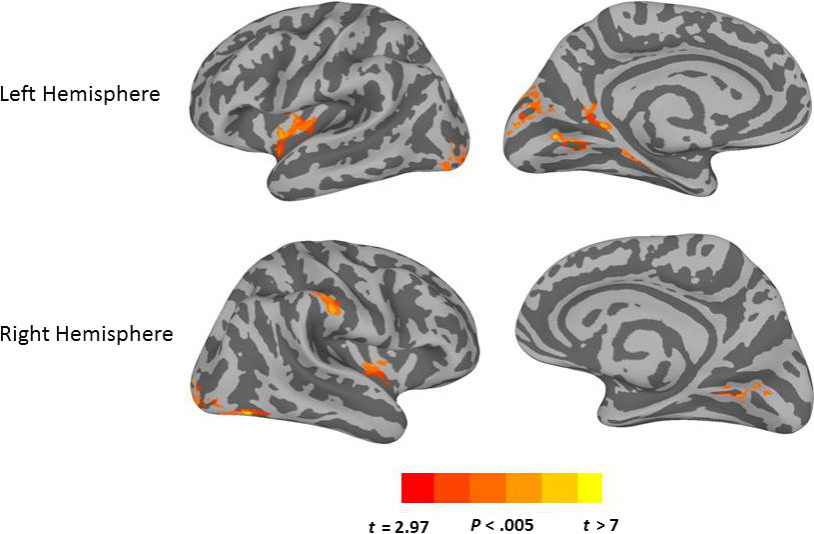
Activation that was common across *Proto* and *Sub* conditions. Cortical surface renderings are overlaid with the results of a conjunction analysis that identified where activation for both *Proto* and *Sub* conditions was jointly significant (*P *<* *0.005, minimum 100 mm^2^ area) during task performance. The figure shows the minimum statistic (significant *t*‐value) from the MSCN analysis encoded in the color bar.

Figure 5 shows the results of a MSCN analysis to identify clusters of commonly activated voxels across the *Proto* and *Sub* conditions. These results are shown with a less conservative single voxel threshold *P *<* *0.005 and a minimum area of 100 mm^2^. Clusters that survive this threshold are sparse and are located in both hemispheres, including the insula, inferior occipital gyrus, cuneus, and lingual gyrus in the left hemisphere, and middle occipital gyrus, fusiform gyrus, insula and IPL in the right hemisphere. This suggests that *Proto* and *Sub* trials, although activating some common neural resources for visual processing, actually recruit disparate neural activity.

**Figure 6:**
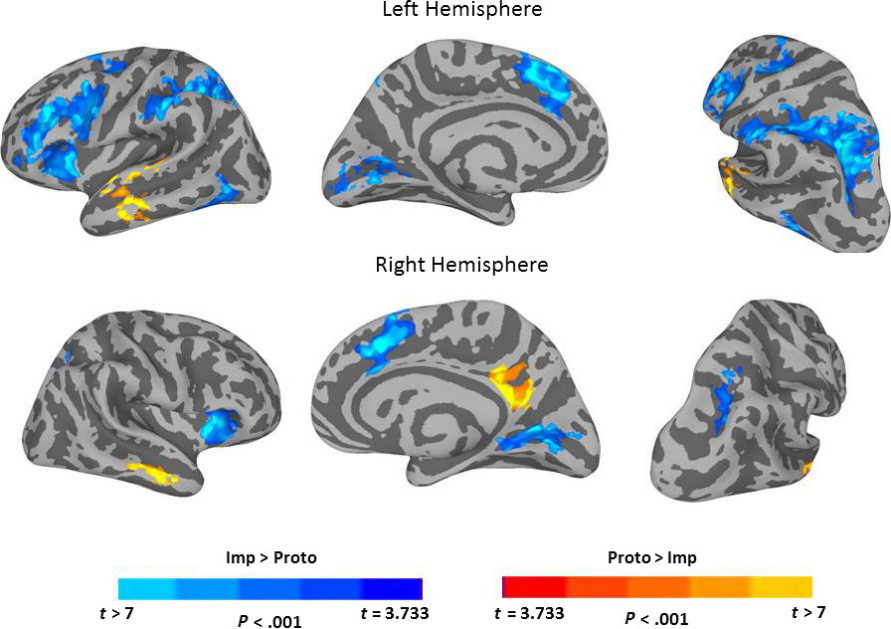
Activation that was different between *Proto* and *Imp* conditions. Cortical surface renderings are overlaid with the results of the contrast *Proto* versus *Imp*. Clusters where activation for *Proto* was stronger are shown in the yellow color scale. Clusters where activation was greater for *Imp* are shown in the blue color scale. The results of this contrast resemble the contrasts shown in Figures 3 and 4. In addition, the right hemisphere was also significantly more strongly activated during the *Imp* trials in the anterior insula, posterior parietal sulcus, medial occipital cortex and supplementary motor area.

Figure 6 shows the results of the contrast for *Proto* versus *Imp* trials at the whole brain cluster corrected *P *<* *0.05 (single voxel *P *<* *0.001). The clusters that were more strongly activated for *Imp* trials (blue color scale) overlap with the same regions shown in Figure 4, suggesting that *Sub* and *Imp* trials engage similar neural resources in the left hemisphere. Although the clusters overlapped, the activation differences were much stronger for this contrast, the clusters were substantially larger (see Table 2), and included an additional cluster in the medial occipital cortex in the left hemisphere. In addition, there were four clusters of stronger activation in the right hemisphere that were not found in the *Sub*>*Proto* contrast, including the lingual gyrus, insula, cingulate gyrus and precuneus. This suggests that participants engaged additional resources above and beyond those recruited by the *Sub* trials, and suggests that they were indeed trying to solve the task. The clusters of activation in the right hemisphere are commonly associated with visual processing and error monitoring/detection (Medford and Critchley 2010; Ullssperger et al. 2010), and may have been recruited either in order to identify a suitable substitute, or to emit a random guess/error response. Moreover, the results of this contrast further support the results shown in Figure 3 in that a mid‐anterior region of both the left and right MTG (and STS/STG) are part of clusters that were more strongly activated for *Proto* trials.

## Discussion

This experiment investigated whether tool selection differently activates the ventral and dorsal stream depending on whether it involves a prototypical tool or a functionally substitutable object. Prototypical tool versus substitute objects differently activated regions of the ventral and dorsal streams previously associated with function and action tool‐related knowledge, respectively (see Figs. 3, 4). Bilateral clusters in the anterior temporal cortex with peak voxels at the MTG were more strongly activated when selecting tools for their prototypical use, and the left MFG, SMA, IPL and IPS were more strongly activated when deciding which object could be used to substitute for the prototypical tool. Moreover, each of these regions tended to show opposing effects in the *Proto* and *Sub* conditions, which was further exaggerated when contrasting *Proto* and *Imp* conditions. In addition, a conjunction analysis revealed that clusters of activation that were present in both conditions included bilateral insula and visual cortex, and the right IPL, but did not include canonical tool regions in the posterior and inferior temporo‐occiptal cortex. Together, these findings parallel the ventral‐dorsal distinction for processing semantic versus structural properties of visual objects (Milner and Goodale 2008), and implies that prototypical tool selection is a subdomain of semantic knowledge processing, and that deciding if an object may be used as a tool for a specific purpose when the action‐goal and recipient are known is a subdomain of action knowledge processing.

Previous research has dissociated the involvement of the IPL and IPS for praxic behaviors such as object manipulation, gesturing, imitation, and tool use, and the MTG for knowledge relating the conventional use of tools and objects to typical recipients of such actions. Activation of the MTG has been reported during tasks that rely on semantic knowledge such as naming tools, understanding tool functions, and recognizing conventional tool‐recipient pairs (Chao et al. 1999; Menz et al., 2010; Mizelle & Wheaton, 2010a,b). In contrast, the IPL and IPS are involved during tasks that rely on object manipulation affordances such as gestures and graspability (Boronat et al. 2005; Buxbaum et al., 2006, Buxbaum et al. 2014), or recognizing familiar tools (Vingerhoets 2008). We found stronger activation in a cluster with a peak at the MTG when choosing a tool for its prototypical use, and stronger activity in the MFG, SMA, IPL, and IPS when deciding if an object could sufficiently substitute for the prototypical tool. Such information cannot be ascertained from the object's semantic properties, and must be derived in an online fashion by considering the motoric affordances, such as graspability and manipulability, as well as the physical properties of the object. In contrast, prototypical tool selection is comparable to a semantic memory‐guided visual search in the context of a particular recipient and action‐goal. If the tool targeted by memory is not available in the visual array, then other processes are engaged to decide if an alternative object may sufficiently substitute for the tool. As such, our findings support the dissociation of action and function tool‐related knowledge with perception and analysis of their structural and semantic properties in dorsal and ventral pathways, respectively.

Because of the fact that the task can be conceptualized as a visual search and analysis of visual perceptual features, as described above, it is possible that task‐related differences in neural activity in the PFC (overlapping with the frontal eye fields) are in fact accounted for by eye movements. However, eye movements are synchronized across both eyes during a visual search task, thereby recruiting activation of the frontal eye fields *bilaterally*. If eye movements were sufficient explanation for our data, then we would have found nearly symmetric activation of the frontal eye field regions across both the left and right hemisphere. We reported two contrasts in which a region of the PFC that overlaps with the frontal eye fields was significantly differently activated, but only *unilaterally*. As such, even in the condition where eye movements and visual search would be maximized (the *Imp* condition, which also had the longest response times), neural activity overlapping the frontal eye field emerged as a significant difference for only one side of the brain. This evidence supports the argument that eye movements do not account for differences in neural activation in our study, and further bolsters our claim that a left lateralized network is involved in the cognitive processes mediating substitutable tool selection. Eye tracking could be used in combination with the tool selection task to disentangle the processing of which visual features contribute to the decision that a particular object can substitute for a prototypical tool, as well as how the neural representation of semantic memory guides visual search for the prototypical tool.

The location of MTG activation that was greater on *Proto* than *Sub* trials was more anterior (Brodamann area [BA] 21) to the canonical tool regions (BA 37, MT/V5) in the posterior MTG/ITG. Activation of the posterior MTG/ITG (BA 37, MT/V5) has been reported for numerous tool‐related perceptual and semantic processing studies, including selective visual processing for tools over animals, as well as naming pictures of tools and discriminating hand‐tool motion (Chao et al. 1999; Chao and Martin 2000; Beauchamp et al. 2002, 2003; Noppeney et al. 2006; Perini et al. 2014). In this experiment, the canonical tool region of the posterior MTG/ITG was not significantly activated. This may be due to the fact that the visual stimulus was more complex than a single tool and included objects, most of which were not typical tools, as well as words, and also did not involve tool or hand motion. As such, prototypical tools, although the subject of a semantic memory guided visual search, were not a dominant visual feature and did not elicit consistent activation in the posterior tool perception regions that was detectable with the *Proto* predictor time course. This further suggests that participants not only did not rely on analysis of the visual features of the tool to make a decision during *Proto* trials, they also did not rely on the name or imagined actions of the prototypical tool, instead guiding visual search by semantic memory supported by the anterior temporal cortex.

The clusters that differentiated the *Proto* and *Sub* conditions had peaks in the MTG, but the cluster was not restricted to the MTG per se, as it extended into the STS and STG. Some studies have reported involvement of the anterior MTG during tool related processing. For example, Mizelle and Wheaton (2010b) showed that EEG source localized activity for tool‐recipient matches and mismatches occurred in a large cluster with a peak in the MTG within about 6‐8 mm of the coordinates reported here for the left hemisphere where *Proto* was more strongly activated than *Sub* trials. But the anterior temporal cortex, including the MTG, STS, and STG, has been implicated more broadly in semantic processing, and there is evidence that tool‐related semantic knowledge also activates STS and STG. Ishibashi et al. (2011)showed that rTMS to the anterior temporal lobe disrupted performance on a tool function similarity judgment task, but not a tool manipulation judgment task. And, Wei et al. (2012) showed that task‐free fluctuations in neural activity in a region of the left MTG more anterior to BA 37 was associated with semantic knowledge of tools. The contrast of *Proto* and *Imp* trials revealed a larger and more distributed cluster of activation that was stronger for *Proto* trials within the left anterior temporal cortex that spanned the MTG, STS, and STG. Thus, the findings of this experiment are in general agreement with the literature concerning tool‐related semantic knowledge processing and activity of the anterior temporal cortex, and suggest that semantic tool‐related knowledge involving the recipient/action‐goal/prototypical tool triad may be specifically related to activity of the anterior MTG.

Only the left hemisphere was significantly more activated for substitutable tool selection than prototypical tool selection. The results of numerous neuroimaging and lesion‐based clinical experiments investigating the neural representation of tool‐related semantic and praxic knowledge, which have utilized a variety of knowledge assessments and stimulation paradigms, generally agree that a left lateralized network involving the parietal cortex is especially important for planning and executing tool use behaviors (Buxbaum and Saffran 2002; Goldenberg & Spatt, 2009; Johnson‐Frey et al. 2005; Kroliczak and Frey 2009; Peeters et al. 2013; Sunderland et al. 2013; Martin et al. 2016). Our results further emphasize the importance of the left hemisphere for tool‐related behavior, and extend them to include a specific role in deciding whether an object may be used as a tool for a specified action. A similar left lateralized brain network incorporating the MFG, SMA, IPL, and IPS may be involved in the development of improved tools, as well as the discovery of novel tools.

The RTs for *Proto* trials were significantly faster than *Sub* trials suggesting that task difficulty could be a confounding explanation with respect to the task differences in neural activity between *Proto* and *Sub* conditions. The PCC, which was more strongly activated for *Proto* trials in comparison to both the *Sub* and *Imp* trials, is a major node of the canonical default mode network (DMN; Buckner et al. 2008). Because activity of the DMN is known to correlate negatively with task difficulty (McKiernan et al. 2003), and the *Proto* condition seems less demanding in terms of processing time, the activation differences in the MTG and PCC could reflect that the DMN was less deactivated by *Proto* trials, as opposed to being directly involved in prototypical tool selection. Trial‐specific RTs for the left and right hand responses were used as parametric predictors in the fMRI analyses, which accounts for some neural activity related to RTs, but it is not known to correlate with the DMN specifically and does not protect altogether against finding differences in neural activity related to task difficulty. However, the canonical DMN regions that are associated with task‐related deactivation include the medial prefrontal cortex (PFC), PCC/precuneus, and lateral inferior parietal cortex, each bilaterally (Buckner et al. 2008), and does not typically include the MTG, which is reportedly anti‐correlated with the PCC at rest (Udin et al. 2009). That we did not find a difference in the medial PFC, together with the left lateralized differences identified by the *Sub* condition and the positive relationship between the *Proto* predictor time course and both the MTG and PCC (i.e., they are not anticorrelated), suggest that the DMN per se was not differently deactivated between conditions, despite differences in RT and difficulty. Importantly, some DMN nodes have been implicated in semantic processing (Binder and Desai 2011; Wirth et al. 2011), and may become involved in certain types of semantic memory‐guided behaviors. With this in mind, finding that some nodes of the DMN, such as the PCC, are involved during prototypical tool selection in combination with more common semantic memory regions in the anterior temporal cortex supports the notion that it is mediated by semantic memory processing. Moreover, the longer RTs for *Sub* trials are consistent with the notion that more attributes (i.e., structural and physical properties, motor affordances) of the visual stimulus need to be processed in order to choose a sufficient substitute. In sum, despite differences in RT, evidence advocates that resources specific to each task condition were identified by the contrasting neural activation rather than a confounding activation difference due to task difficulty.

The *Sub* condition may also access additional resources that are not specific to tool‐related knowledge per se, such as working memory (i.e., task‐positive network) or decision mechanisms. The experiment was designed to investigate whether decisions about the usability of an object as a tool would recruit similar or different neural activation than decisions about which tool is the prototypical tool for a specific purpose. This aim of the experiment was achieved in that we found that choosing a prototypical tool and deciding which object can be used as a tool to substitute for the prototypical tool when it is unavailable did indeed recruit different neural activation pathways. The activation during the *Sub* condition activated regions associated with tool‐specific motoric knowledge (i.e., action knowledge) in the dorsal processing stream, which unavoidably overlaps with the working memory system in the left hemisphere. We interpret this to indicate that tool‐related knowledge is activated, and being operated on by the working memory system in order to make a decision within the demands of the task. This is consistent with the design of the task in that a functionally substitutable object requires online processing of its motoric affordances (i.e., action knowledge) in conjunction with representations of the task goal and recipient object – which may require integration via the working memory system. Future research may aim to disentangle the neural activity associated with each of these component processes.

While the evidence from this study supports the conclusion that *Proto* and *Sub* tool selection differently activate the ventral and dorsal streams because they rely on function and action knowledge, respectively, other findings in the literature report finding tool‐related action knowledge represented in the ventral stream in the posterior MTG/ITG (Perini et al. 2014), as well as encroaching on more anterior regions of the MTG and temporal cortex than the canonical tool regions (Buxbaum et al. 2014). However, the study by Perini et al. (2014) presented a single tool image on each trial and involved only analyses of neuroimaging data in localizer‐identified (tools>animals) regions‐of‐interest (ROIs) that were defined a priori (whole‐brain analyses were not reported), and contrasted knowledge of a tool's typical gesture as action knowledge, with its typical location (i.e., the place you find/store/use that tool) as semantic knowledge, as opposed to function knowledge per se. As such, it is not clear whether this study would have also found differences between action and function knowledge in more anterior regions of the MTG and temporal cortex. Buxbaum et al. (2014) examined tool‐related knowledge motoric abilities, such as gestures and imitations, in patients with left hemisphere lesions due to stroke. They reported that lesions of the MTG (and ventral stream) were related to poorer performance for tool gestures, suggesting a role for ventral stream in action knowledge. However, many of the lesions were diffuse across the left hemisphere and could also include white matter pathways that impair information integration between remote regions during task performance. All tasks required a praxic movement as the dependent variable in response to a visually presented movement stimulus. Because tool‐related motion perception processing is associated with posterior MTG (BA37, MT/V5), impaired gesture, imitation and other tool‐related motor movements by their patients may reflect poor processing of the sample stimulus (gesture for tool), rather than actually being reliant on the MTG or anterior temporal cortex for retrieval of action knowledge.

The clinical neuropsychology literature concerning apraxia documents numerous deficits in various aspects of goal‐directed tool‐use behavior (Heilman 2010). Patients with conceptual apraxia, for example, reportedly fail to accomplish tool‐use action goals either due to incorrect tool selection despite executing or pantomiming the correct motor action, or due to improper execution of the motor action despite correctly selecting the prototypical tool (Ochipa et al. 1989; Heilman et al. 1997; Buxbaum et al. 2000). Despite improper tool selection the action goal may yet be accomplished (Buxbaum et al. 2000) because the selected tool is functionally substitutable. Furthermore, some patients also show deficits in the ability to plan and/or execute multi‐step action sequences that require the use of different tools at each step, and is attributed to interactions of several networks comprising the frontal, temporal and parietal cortices (see Bienkiewicz et al. 2014, for a review), which overlaps with the regions identified in the contrasts of the *Sub* and *Proto* conditions. Further refinement of the tool‐selection task used in this experiment may lead to the development of a useful clinical assessment to dissociate these deficits, as well as for rehabilitation training, and further neuroimaging studies may reveal how the two brain systems of tool‐related knowledge interact during goal‐directed tool‐use behavior.

### Summary

This experiment employed a novel tool selection task to study the relative involvement of the ventral and dorsal streams when choosing an object to use as a tool for a specific purpose. Our findings demonstrate that tool selection may be mediated by either the ventral or dorsal pathway depending on whether it involves selection of the prototypical tool or a functional substitutable object for accomplishment of a specific action‐goal. Prototypical tool selection is related to activity in the ventral stream representing function tool‐related knowledge in the MTG and anterior temporal lobes, and suggests that it is a subdomain of rapidly accessible semantic knowledge. Choosing an alternative object to substitute requires the online perceptual analysis of the structural properties and motor affordances of the visual object, which is slower and more demanding than retrieving semantic knowledge for the prototypical tool, and is related to left lateralized activity in the dorsal stream representing action knowledge in the MFG, SMA, IPL and IPS. Additional neuroimaging research, in combination with eye tracking and other complementary methods, may elucidate how networks representing semantic function knowledge and motoric action knowledge interact during proper tool use.

## Conflict of Interest

None declared.
